# Allelochemicals Identified From Sweet Potato (*Ipomoea batatas*) and Their Allelopathic Effects on Invasive Alien Plants

**DOI:** 10.3389/fpls.2022.823947

**Published:** 2022-04-12

**Authors:** Shicai Shen, Guangzong Ma, Gaofeng Xu, Diyu Li, Guimei Jin, Shaosong Yang, David Roy Clements, Aidong Chen, Lina Wen, Fudou Zhang, Min Ye

**Affiliations:** ^1^Key Laboratory of Prevention and Control of Biological Invasions, Ministry of Agriculture and Rural Affairs of China, Agricultural Environment and Resource Research Institute, Yunnan Academy of Agricultural Sciences, Kunming, China; ^2^Key Laboratory of Green Prevention and Control of Agricultural Transboundary Pests of Yunnan Province, Agricultural Environment and Resource Research Institute, Yunnan Academy of Agricultural Sciences, Kunming, China; ^3^State Key Laboratory for Conservation and Utilization of Bio-Resources in Yunnan, Yunnan Agricultural University, Kunming, China; ^4^Department of Biology, Trinity Western University, Langley, BC, Canada

**Keywords:** sweet potato, allelochemicals, allelopathic effects, invasive plants, weed management

## Abstract

Sweet potato [*Ipomoea batatas* (L.) Lam] is grown as important cash and food crop worldwide and has been shown to exhibit allelopathic effects on other plants. However, its metabolome has not been studied extensively, particularly with respect to the production of phytotoxic bioactive secondary products. In this study, the chemical composition of petroleum ether extract of sweet potato was characterized, and the morphological and physiological effects of some individual components against four invasive alien weeds *Bidens pilosa* L., *Galinsoga parviflora* Cav., *Lolium multiflorum* Lam., and *Phalaris minor* Retz. were determined. Twenty-one components were identified by GS-MS, constituting 96.08% of petroleum ether extract in sweet potato. The major components were palmitic acid (PA) (17.48%), ethyl linoleate (EL) (13.19%), linoleic acid (LA) (12.55%), ethyl palmitate (EP) (11.77%), ethyl linolenate (ELL) (8.29%) oleic acid (5.82%), ethyl stearate (4.19%), and 3-methylphenol acetate (3.19%). The five most abundant compounds exhibited strong inhibition activity against the four invasive weeds tested. The highest inhibition rates were seen for LA, followed by PA and EP, respectively. Catalase (CAT), malondialdehyde (MDA), and peroxidase (POD) content of *L. multiflorum* were increased by the three allelochemicals, i.e., LA, PA and EP, but superoxide dismutase (SOD), chlorophyll-a and chlorophyll-b levels declined. Overall, the combined impact of all five compounds could be quite effective in suppressing the invasive weeds of concern.

## Introduction

Weeds have been considered an important yield limiting factor in agricultural production, as they affect the yield and quality of crops through competition for nutrients, light, water, and space (Chauhan and Johnson, 2011). Chemical herbicides are widely used to prevent and control weed infestations due to their ready availability, low costs, and minimal labor requirements (Grisi et al., 2015). However, excessive use of synthetic herbicides has adversely affected environments, biodiversity, fisheries, and human and animal health, as well as causing soil erosion and resulting in herbicide-resistant weeds (Kong et al., 2006; Bastiaans et al., 2008). To ameliorate these issues, it is necessary to find other environmentally friendly and economically sustainable solutions to existing and emerging weeds infestation problems. A promising weed control option is the potential for exploiting the weed-suppressing ability of crop cultivars themselves.

As a safe and sustainable alternative, plant allelopathy has been widely explored to develop sustainable agricultural systems (Duke, 2007; Macías et al., 2019). A large variety of secondary metabolites have been identified in plants, and many of those compounds exhibit strong allelopathic activities inhibiting seed germination and growth of other plants (Duke et al., 2000; Macías et al., 2019). Many wild and cultivated crops with allelopathic potential have been suggested to potentially suppress weeds but not all are equally effective. Differences in allelopathic potential among crops result from different types and concentrations of allelopathic chemicals that different crop varieties produce and release (Kong et al., 2002). Moreover, commercially acceptable allelopathic crop varieties have been bred and applied, incorporating them into more systems for reducing herbicide use (Kong et al., 2011; Kong, 2018). Therefore, finding more crop germplasm resources with allelopathic activity and identifying related phytotoxic metabolites may be helpful in developing weed management options for sustainable agriculture.

Sweet potato [*Ipomoea batatas* (L.) Lam.: Convolvulaceae], a herbaceous and perennial vine, originated in Central and South America. It is the world’s seventh most important food crop and is widely grown in temperate, subtropical and tropical regions in more than 100 countries (Jung et al., 2011). Sweet potato is widely used as a vegetable, in beverages, for medicines, or as animal feed. This plant is suitable for a wide range of growing conditions and can tolerate drought, flooding, and nutrient deficiencies. Sweet potato can give higher yield with low inputs and less crop management (Ewell and Mutuura, 1994). It is well suited for phytoremediation applications to clean up land contaminated with harmful pollutants and soil erosion protection because of its fast growing and extensive root system (de Araujo et al., 2004). Furthermore, this crop tends to tolerate diseases and pest infestation, especially weeds.

Sweet potato exhibits prolific asexual reproduction, rapid growth, and readily forms extensive canopies in farming systems (Shen et al., 2015). The rapidly spreading sweet potato foliage can cover the field completely and significantly inhibit many weeds. Therefore, sweet potato has been recognized as a competitive crop against various weeds. It significantly suppresses plant morphological and physiological characteristics of some invasive alien weeds (Shen et al., 2015, 2019), and reduces the density of other weed species under field conditions (Shen et al., 2014). Moreover, studies have also demonstrated significant allelopathic activity of sweet potato on various plants with phenolic secondary metabolites associating with chemical interference (Chon and Boo, 2005; Xuan et al., 2012; Shen et al., 2017b,^2018^). However, the metabolites of sweet potato have not been studied extensively, particularly with respect to the production of phytotoxic bioactive secondary products.

In sweet potato fields in our region, four common invasive plant species are *Bidens pilosa* L., *Galinsoga parviflora* Cav., *Lolium multiflorum* Lam., and *Phalaris minor* Retz. *Bidens pilosa* and *G. parviflora* (Asteraceae family) originated in tropical United States, and *L. multiflorum*, and *P. minor* (Poaceae family) are native to Europe (Shen et al., 2017a). They are common weeds in most temperate, subtropical, and tropical regions of Yunnan Province (Shen et al., 2017a). The objectives of this study were to identify the allelochemicals of sweet potato and examine their bioactivity on these invasive alien plants in Yunnan Province, Southwest China. These findings are important to elucidate allelopathy mechanisms in sweet potato-based cropping systems and facilitate exploration of bio-herbicidal or phytotoxic plant secondary metabolites for offering enhanced weed management opportunities.

## Materials and Methods

### Study Species

Sweet potato mainly reproduces by clonal means or seed, with 20–50 cm fragments with 3–5 nodes typically planted (Sihachakr et al., 1997). Since 2011, various sweet potato cultivars in Yunnan Province have been collected and grown in the greenhouse of the Agricultural Environment and Resource Research Institute, Yunnan Academy of Agricultural Sciences, China. Seeds from local populations of four invasive alien plant species (*B. pilosa*, *G. parviflora*, *L. multiflorum*, and *P. minor*) were collected in September 2019 and 2020, dried at room temperature and then kept at −4°C.

### Collection and Extraction of Materials

About 1,500 g fresh aerial parts of the sweet potato plants were collected from the greenhouse of the Agricultural Environment and Resource Research Institute, Yunnan Academy of Agricultural Sciences, China, in September 2020. The parts were washed several times to get rid of soil particles with distilled water and then cut into pieces (approximately 1-3 mm) for extraction of components. Each sample was separately placed in a conical flask (4 L) and distilled water was added. The filtered aqueous extract was subsequently partitioned with various polar solvents including petroleum ether, ethyl acetate, and n-butanol alcohol.

### Identification of Allelochemicals

Bioassays using four (aqueous, petroleum ether, ethyl acetate, and n-butanol alcohol) extracts were examined in a previous study (Ma et al., 2022), and found that petroleum ether extract showed the stronger inhibitory effect. So, the petroleum ether extract was used for identification and quantification of allelopathic compounds in current study. GC-MS was performed using an Agilent 7890 gas chromatograph equipped with a quadrupole mass spectrometer (Agilent 5975 N, United States). A 1 μL aliquot of each sample was injected into an HP–5MS fused silica capillary column (0.25 mm × 30 m i.d., film thickness 0.25 μm) with helium carrier gas flow rate at 1.0 mL/min in the splitless mode. After injection, the oven temperature was programmed to increase from 40 to 80°C at 3°C/min, and from 80 to 280°C at 5°C/min, and then held for 30 min. The ion source temperature was maintained at 230°C, the injector temperature at 280°C, and the quadrupole temperature at 150°C. The mass spectrometer was fitted with an EI source operated at 70 eV and mass spectra were recorded in the range from 50 to 500 *m/z.* Injections were made using the split mode of 5:1. Their mass spectra were compared with those of authentic standards from the NIST 08 library with more than 90% matching values.

Five main standard compounds, palmitic acid (PA), ethyl linoleate (EL), linoleic acid (LA), ethyl palmitate (EP), and ethyl linolenate (ELL) were selected for further GC-MS analysis. All chemicals were purchased from Aladdin Biochemical Technology Co., Ltd., Shanghai, China as high-purity standards. Standard compounds were chromatographed alone and as mixtures. Retention times for the standard compounds and the major peaks in the extract were recorded. Chemical compounds from each fraction were identified by retention times or standard addition, and their amounts were calculated by comparing peak area with those of standards.

### Bioassay and Physiological Activities of Allelochemicals

According to the results of the “Identification of Components from Petroleum Ether Extract,” five main commercial compounds PA, EL, LA, EP, and ELL were selected for further bioassay experiments, which accounted for the highest relative contents and higher matching value of all identified components. The seeds of four invasive weeds, *B. pilosa*, *G. parviflora*, *L. multiflorum*, and *P. minor* were used to test the morphological inhibitory effects of these components. Five extract concentrations (0.125, 0.25, 0.5, 1.0, and 2.0 mg mL^–1^) and a control (distilled water) (CK) were used in this experiment for each compound. In each treatment, 10 sterile seeds of each of the four invasive weed species were evenly placed in a separate petri dishes, and 1 mL of extract or distilled water (control) were added. The culture conditions were the same as the above bioassay experiment, and the experiment had four replications. After 7 days, samples were collected to determine the germination, shoot height, root length, and fresh biomass.

Based on the bioassay of the above five components, the physiological effects of three stronger allelechemicals PA, EP, and LA on *L. multiflorum* were further measured. The levels of antioxidant enzymes superoxide dismutase (SOD), catalase (CAT), peroxidase (POD), malondialdehyde (MDA), chlorophyll-a, and chlorophyll-b of PA, EP, and LA in *L. multiflorum* seedlings were tested and analyzed in the laboratory at the Agricultural Environment and Resources Research Institute of Yunnan Academy of Agricultural Sciences (Zhang et al., 2016; Cheng et al., 2018).

### Data Analyses

The allelopathic response index (IR; Williamson and Richardson, 1988) for germination, plant height and biomass was calculated using the following formulas: when T ≥ C, RI = 1-C/T; when T < C, RI = T/C-1 (T < C). C is the control value and T is the treatment value, RI > 0 represents a stimulatory effect, RI < 0 represents an inhibitory effect, and the absolute value is consistent with the allelopathy intensity. Data was analyzed by analysis of variance (one-way ANOVA). If significant differences were detected by ANOVA, Duncan’s multiple range tests were used to detect differences among treatments at a 5% level of significance.

## Results

### Identification of Allelopathic Substances

**FIGURE 1:**
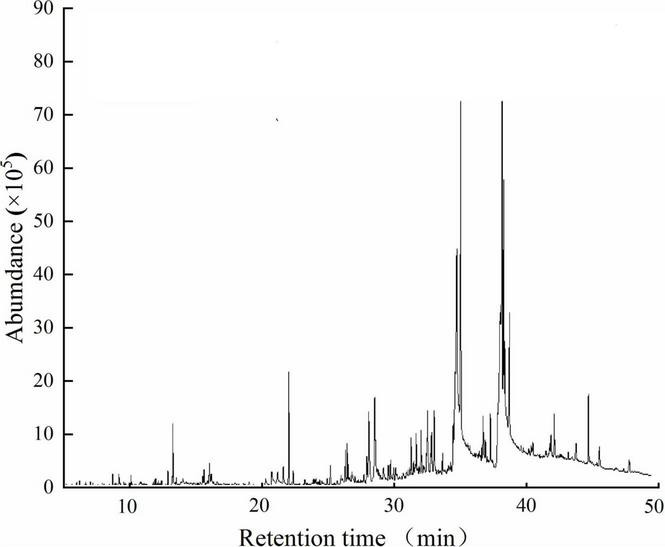
Total ion flow graph of sweet potato petroleum ether extract.

**FIGURE 2:**
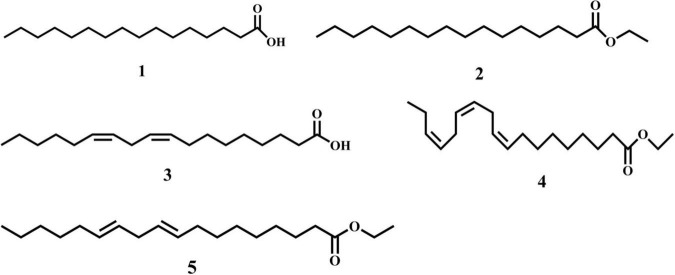
Structures of five major components found in sweet potato petroleum ether extract: (1) palmitic acid; (2) ethyl palmitate; (3) linoleic acid; (4) ethyl linolenate; (5) ethyl linoleate. The numbers of individually tested components are underlined.

Twenty-one components representing 96.08% of the sweet potato petroleum ether extract were identified using GC–MS (Figure 1 and Table 1). The major components were PA (17.48%), EL (13.19%), LA (12.55%), EP (11.77%), ELL (8.29%), oleic acid (5.82%), ethyl stearate (4.19%), and 3-methylphenol acetate (3.19%) (Table 1). The five largest components: PA, EL, LA, EP, and ELL were confirmed and consistent with standard commercially purchased compounds through GC-MS. The chemical structures of the five major components in sweet potato are shown in Figure 2. However, the allelopathic potential of these components on invasive alien plants has not been reported before.

**TABLE 1:** Chemical compounds of sweet potato petroleum ether extract.

No.	Compound	Retention time (min)	Percentage (%)	Molecular ion (m/z)	Main fragment ions (m/z)
1	Linalool	14.282	1.32	154	71 (99.9); 93 (60.9); 41 (56.9); 69 (48.2); 43 (48.2); 55 (45.0); 80 (26.2); 121 (17.3); 83 (16.1); 67 (15.9)
2	Isocaryophyllene	22.942	2.18	204	41 (99.9); 93 (77.9); 69 (73.0); 79 (51.9); 91 (50.6); 133 (50.5); 81 (36.0); 39 (34.3); 105 (33.9); 107 (32.6)
3	spathulenol	27.169	0.63	220	43 (99.9); 41 (68.8); 91 (42.2); 119 (42.1); 93 (39.7); 69 (34.9); 105 (34.1); 159 (33.5); 205 (33.2); 79 (31.6)
4	Caryophyllene oxide	27.298	0.67	220	41 (99.9); 43 (66.6); 79 (47.3); 93 (44.5); 91 (41.8); 55 (30.8); 69 (27.4); 107 (25.4); 67 (25.3); 105 (24.9)
5	3-methylphenol acetate	28.908	3.19	150	108 (99.9); 43 (42.1); 150 (35.7); 107 (15.2); 109 (14.9); 49 (10.2); 77 (9.4); 84 (8.3); 79 (7.9); 44 (7.1)
6	3,5,5-trimethyl-4-(3-oxobutyl)cyclohex-2-en-1-one	29.353	2.96	208	151 (99.9); 135 (82.7); 43 (81.9); 109 (74.6); 95 (41.4); 208 (40.5); 123 (27.9); 138 (26.3); 41 (24.3); 108 (21.0)
7	Hexahydrofarnesyl acetone	32.821	0.98	268	43 (99.9); 58 (89.8); 71 (45.0); 57 (42.6); 59 (40.7); 41 (37.3); 55 (34.5); 69 (24.5); 85 (22.9); 95 (20.3)
8	2-isopropyl-4a-methyl-8-methylenedecahydro-1,5-naphthalenediol	33.301	2.37	238	220 (99.9); 107 (82.0); 40 (79.0); 121 (68.0); 81 (62.0); 29 (62.0); 79 (58.0); 93 (58.0); 109 (54.0); 123 (54.0)
9	(1aS,3aS,6aS,6bR)-5,5,6b-trimethyl-3a,4,6,6a-tetrahydro-1H-cyclopropa[e]indene-1a,2-dicarbaldehyde	33.61	1.59	232	43 (99.9); 55 (84.0); 93 (73.9); 41 (69.7); 232 (67.8); 91 (66.4); 105 (64.8); 131 (63.8); 53 (61.6); 79 (59.9)
10	Boronal	33.807	1.97	252	173 (99.9); 145 (90.9); 105 (81.9); 41 (79.0); 81 (73.5); 69 (73.1); 43 (71.7); 234 (69.5); 91 (67.0); 93 (59.3)
11	Dibutyl phthalate	35.219	1.17	278	149 (99.9); 41 (21.5); 43 (14.5); 150 (10.2); 57 (7.7); 55 (7.0); 56 (6.2); 76 (5.2); 223 (4.6); 42 (4.6)
12	Palmitic acid	35.49	17.48	256	43 (99.9); 73 (90.5); 60 (83.8); 41 (74.9); 57 (63.4); 55 (61.6); 29 (41.1); 69 (31.0); 71 (28.5); 61 (21.8)
13	Ethyl palmitate	35.781	11.77	284	88 (99.9); 101 (55.9); 43 (36.2); 41 (26.8); 55 (23.3); 29 (22.7); 57 (21.7); 73 (15.6); 89 (13.6); 69 (13.2)
14	Phytol	38.003	1.09	296	71 (99.9); 43 (78.6); 57 (66.2); 41 (65.1); 55 (59.0); 69 (51.4); 81 (47.0); 68 (43.7); 82 (36.1); 95 (32.4)
15	Linoleic acid	38.738	12.55	280	67 (99.9); 55 (95.4); 81 (79.3); 41 (70.8); 69 (56.0); 95 (54.5); 68 (53.0); 54 (48.6); 43 (48.0); 82 (47.9)
16	Ethyl linoleate	38.871	13.19	308	67 (99.9); 81 (91.9); 81 (77.2); 41 (70.7); 95 (68.3); 68 (61.3); 82 (56.9); 54 (52.5); 96 (47.8); 69 (44.8)
17	Ethyl linolenate	39.000	8.29	306	79 (99.9); 67 (62.7); 95 (60.0); 93 (52.3); 55 (46.3); 81 (44.4); 108 (42.5); 80 (40.9); 41 (40.4); 91 (30.5)
18	Oleic acid	39.077	5.82	282	55 (99.9); 69 (75.7); 41 (75.2); 83 (59.3); 43 (54.9); 97 (44.3); 57 (43.9); 67 (35.3); 56 (34.5); 70 (32.0)
19	Ethyl stearate	39.422	4.19	312	88 (99.9); 101 (65.6); 43 (45.2); 55 (40.9); 41 (35.9); 57 (31.3); 89 (18.1); 69 (15.8); 73 (15.4); 70 (13.6)
20	Ethyl icosanoate	42.771	1.02	340	88 (99.9); 101 (64.0); 43 (40.8); 55 (27.0); 41 (25.3); 57 (25.0); 29 (23.4); 69 (16.2); 73 (15.8); 89 (11.7)
21	Bis(2-ethylhexyl) phthalate	45.328	1.66	391	149 (99.9); 167 (35.0); 57 (34.1); 70 (26.4); 41 (22.5); 71 (22.4); 55 (21.8); 43 (20.0); 150 (10.7); 83 (10.0)

### Allelopathic Effects of Five Components on the Growth of Four Invasive Weeds

The results showed that the germination and seedling growth of four invasive weeds, *B. pilosa*, *G. parviflora*, *L. multiflorum*, and *P. minor* were significantly affected by five components of the extract: PA, EL, LA, EP, and ELL. Apart from the shoot length of *B. pilosa* at a concentration of 0.125 mg mL^–1^ of PA, germination rate, root length, shoot length, and biomass of all four invasive weeds were significantly reduced with increasing concentration of these five components (Tables 2, 3). Generally invasive weed biomass and root length were most strongly inhibited, followed by shoot length and finally germination rate was the least affected. The highest inhibition rates were seen for LA, followed by PA and EP, with the lowest inhibition rates seen for EL and ELL (Tables 2, 3).

**TABLE 2:** Effects of five components of sweet potato ether petroleum extract on the growth of *Lolium multiflorum* and *Phalaris minor*.

Compound	Concentration/(mg mL^–1^)	Response index							
		*Lolium multiflorum*				*Phalaris minor*			
		Germination	Root length	Shoot length	Biomass	Germination	Root length	Shoot length	Biomass
Palmitic acid	0.125	−0.14 ± 0.03a	−0.31 ± 0.04a	−0.29 ± 0.02a	−0.39 ± 0.03a	−0.14 ± 0.06a	−0.48 ± 0.03a	−0.03 ± 0.04a	−0.43 ± 0.10a
	0.25	−0.17 ± 0.00ab	−0.38 ± 0.04a	−0.44 ± 0.01b	−0.55 ± 0.02b	−0.21 ± 0.07a	−0.51 ± 0.02ab	−0.05 ± 0.01a	−0.72 ± 0.01b
	0.50	−0.28 ± 0.06bc	−0.41 ± 0.02a	−0.41 ± 0.05b	−0.57 ± 0.03b	−0.29 ± 0.04a	−0.60 ± 0.01b	−0.29 ± 0.01b	−0.77 ± 0.04b
	1.00	−0.38 ± 0.06c	−0.60 ± 0.06b	−0.77 ± 0.04c	−0.85 ± 0.02c	−0.46 ± 0.06b	−0.73 ± 0.05c	−0.37 ± 0.03c	−0.84 ± 0.01b
	2.00	−1.00 ± 0.00d	−1.00 ± 0.00c	−1.00 ± 0.00d	−1.00 ± 0.00d	−1.00 ± 0.00c	−1.00 ± 0.00d	−1.00 ± 0.00d	−1.00 ± 0.00c
Ethyl	0.125	−0.21 ± 0.03a	−0.19 ± 0.04a	−0.16 ± 0.02a	−0.30 ± 0.03a	−0.14 ± 0.06a	−0.63 ± 0.06a	−0.10 ± 0.04a	−0.52 ± 0.02a
palmitate	0.25	−0.24 ± 0.03ab	−0.19 ± 0.02a	−0.25 ± 0.03b	−0.39 ± 0.02ab	−0.18 ± 0.04a	−0.67 ± 0.10ab	−0.09 ± 0.08a	−0.73 ± 0.03b
	0.50	−0.31 ± 0.03b	−0.32 ± 0.09a	−0.33 ± 0.04b	−0.50 ± 0.07b	−0.25 ± 0.06a	−0.66 ± 0.12ab	−0.20 ± 0.13a	−0.81 ± 0.04c
	1.00	−0.52 ± 0.03c	−0.53 ± 0.06b	−0.61 ± 0.01c	−0.78 ± 0.03c	−0.43 ± 0.07b	−0.76 ± 0.06ab	−0.46 ± 0.03b	−0.88 ± 0.02cd
	2.00	−1.00 ± 0.00d	−1.00 ± 0.00c	−1.00 ± 0.00d	−1.00 ± 0.00d	−0.82 ± 0.04c	−0.88 ± 0.02b	−0.77 ± 0.03c	−0.91 ± 0.00d
Linoleic acid	0.125	−0.17 ± 0.06a	−0.24 ± 0.04a	−0.31 ± 0.03a	−0.45 ± 0.01a	−0.25 ± 0.06a	−0.48 ± 0.01a	−0.11 ± 0.01a	−0.27 ± 0.09a
	0.25	−0.48 ± 0.06b	−0.46 ± 0.02b	−0.45 ± 0.05b	−0.73 ± 0.04b	−0.29 ± 0.04a	−0.63 ± 0.08b	−0.40 ± 0.03b	−0.55 ± 0.04a
	0.50	−0.52 ± 0.03b	−0.54 ± 0.05b	−0.52 ± 0.06b	−0.79 ± 0.04b	−0.50 ± 0.04b	−0.91 ± 0.03c	−0.58 ± 0.02c	−0.66 ± 0.03b
	1.00	−0.62 ± 0.09b	−0.79 ± 0.02c	−0.81 ± 0.03c	−0.94 ± 0.02c	−0.64 ± 0.04c	−0.90 ± 0.01c	−0.69 ± 0.01d	−0.90 ± 0.02b
	2.00	−1.00 ± 0.00b	−1.00 ± 0.00d	−1.00 ± 0.00d	−1.00 ± 0.00c	−1.00 ± 0.00d	−1.00 ± 0.00c	−1.00 ± 0.00e	−1.00 ± 0.00c
Ethyl	0.125	−0.07 ± 0.00a	−0.24 ± 0.05a	−0.18 ± 0.02a	−0.24 ± 0.04a	−0.11 ± 0.04a	−0.48 ± 0.00a	−0.03 ± 0.01a	−0.03 ± 0.04a
linolenate	0.25	−0.24 ± 0.07b	−0.25 ± 0.03a	−0.24 ± 0.05ab	−0.32 ± 0.04a	−0.29 ± 0.09b	−0.49 ± 0.01ab	−0.04 ± 0.03a	−0.12 ± 0.04a
	0.50	−0.31 ± 0.03bc	−0.30 ± 0.01a	−0.24 ± 0.03ab	−0.45 ± 0.02b	−0.29 ± 0.04b	−0.53 ± 0.01b	−0.12 ± 0.07a	−0.36 ± 0.06b
	1.00	−0.41 ± 0.03c	−0.47 ± 0.05b	−0.37 ± 0.08bc	−0.64 ± 0.02d	−0.21 ± 0.04ab	−0.61 ± 0.02c	−0.34 ± 0.04b	−0.63 ± 0.06c
	2.00	−0.66 ± 0.03d	−0.63 ± 0.03c	−0.49 ± 0.05c	−0.94 ± 0.01d	−0.29 ± 0.04b	−0.70 ± 0.02d	−0.55 ± 0.03c	−0.64 ± 0.07c
Ethyl	0.125	−0.14 ± 0.03a	−0.10 ± 0.02a	−0.12 ± 0.03a	−0.32 ± 0.03a	−0.14 ± 0.00a	−0.54 ± 0.03a	−0.13 ± 0.03a	−0.04 ± 0.03a
linoleate	0.25	−0.28 ± 0.06ab	−0.27 ± 0.03b	−0.27 ± 0.04ab	−0.47 ± 0.04b	−0.14 ± 0.06a	−0.62 ± 0.11ab	−0.25 ± 0.08a	−0.26 ± 0.07b
	0.50	−0.31 ± 0.03b	−0.31 ± 0.05b	−0.30 ± 0.06b	−0.53 ± 0.02b	−0.25 ± 0.00ab	−0.69 ± 0.02ab	−0.18 ± 0.02a	−0.54 ± 0.09c
	1.00	−0.21 ± 0.03ab	−0.31 ± 0.03b	−0.40 ± 0.01b	−0.71 ± 0.01c	−0.32 ± 0.04b	−0.76 ± 0.06bc	−0.23 ± 0.06a	−0.64 ± 0.01cd
	2.00	−0.48 ± 0.06c	−0.70 ± 0.02c	−0.60 ± 0.07c	−0.85 ± 0.03d	−0.36 ± 0.06b	−0.81 ± 0.01c	−0.57 ± 0.02b	−0.78 ± 0.05d

*Data are expressed as mean ± standard deviation. Different letters within the same column under same compound signify significant differences at P < 0.05.*

**TABLE 3:** Effects of five components of sweet potato ether petroleum extract on the growth of *Bidens pilosa* and *Galinsoga parviflora*.

Compound	Concentration/(mg mL^–1^)	Response index							
		*Bidens pilosa*				*Galinsoga parviflora*			
		Germination	Root length	Shoot length	Biomass	Germination	Root length	Shoot length	Biomass
Palmitic acid	0.125	−0.10 ± 0.03a	−0.05 ± 0.05a	0.00 ± 0.03a	−0.46 ± 0.02a	−0.24 ± 0.03a	−0.11 ± 0.03a	−0.32 ± 0.09a	−0.54 ± 0.08a
	0.25	−0.17 ± 0.06ab	−0.48 ± 0.02b	−0.29 ± 0.08b	−0.51 ± 0.03a	−0.31 ± 0.07a	−0.18 ± 0.04a	−0.53 ± 0.03b	−0.67 ± 0.02ab
	0.50	−0.24 ± 0.03bc	−0.64 ± 0.05c	−0.42 ± 0.05b	−0.68 ± 0.04b	−0.34 ± 0.07a	−0.66 ± 0.01b	−0.66 ± 0.01b	−0.76 ± 0.05b
	1.00	−0.31 ± 0.03c	−0.74 ± 0.03c	−0.60 ± 0.01c	−0.74 ± 0.04b	−0.38 ± 0.06a	−0.82 ± 0.02c	−0.82 ± 0.02c	−0.77 ± 0.03b
	2.00	−1.00 ± 0.00d	−1.00 ± 0.00d	−1.00 ± 0.00cd	−1.00 ± 0.00c	−1.00 ± 0.00b	−1.00 ± 0.00d	−1.00 ± 0.00d	−1.00 ± 0.00c
Ethyl	0.125	−0.24 ± 0.07a	−0.03 ± 0.04a	−0.11 ± 0.03a	−0.41 ± 0.03a	−0.14 ± 0.03a	−0.05 ± 0.03a	−0.22 ± 0.04a	−0.55 ± 0.03ab
palmitate	0.25	−0.21 ± 0.03a	−0.32 ± 0.05b	−0.20 ± 0.03a	−0.52 ± 0.04a	−0.17 ± 0.00a	−0.21 ± 0.07b	−0.37 ± 0.08b	−0.52 ± 0.07a
	0.50	−0.41 ± 0.07b	−0.49 ± 0.01c	−0.41 ± 0.05b	−0.69 ± 0.04b	−0.31 ± 0.03b	−0.30 ± 0.05b	−0.61 ± 0.05c	−0.68 ± 0.04bc
	1.00	−0.48 ± 0.06b	−0.63 ± 0.03d	−0.56 ± 0.03c	−0.74 ± 0.06b	−0.38 ± 0.06b	−0.68 ± 0.03c	−0.81 ± 0.03d	−0.75 ± 0.05c
	2.00	−1.00 ± 0.00c	−1.00 ± 0.00e	−1.00 ± 0.00d	−1.00 ± 0.00c	−1.00 ± 0.00c	−1.00 ± 0.00d	−1.00 ± 0.00e	−1.00 ± 0.00d
Linoleic acid	0.125	−0.14 ± 0.03a	−0.24 ± 0.03a	−0.17 ± 0.02a	−0.46 ± 0.02a	−0.17 ± 0.00a	−0.19 ± 0.05a	−0.42 ± 0.05a	−0.64 ± 0.06a
	0.25	−0.24 ± 0.03b	−0.49 ± 0.05b	−0.39 ± 0.02b	−0.58 ± 0.05b	−0.21 ± 0.09ab	−0.43 ± 0.04b	−0.70 ± 0.05b	−0.69 ± 0.04a
	0.50	−0.34 ± 0.03c	−0.51 ± 0.04b	−0.42 ± 0.02b	−0.66 ± 0.01bc	−0.28 ± 0.06ab	−0.61 ± 0.04c	−0.78 ± 0.02bc	−0.69 ± 0.03a
	1.00	−0.45 ± 0.03d	−0.76 ± 0.03c	−0.67 ± 0.03c	−0.74 ± 0.04c	−0.34 ± 0.03b	−0.80 ± 0.02d	−0.88 ± 0.01c	−0.84 ± 0.03b
	2.00	−1.00 ± 0.00e	−1.00 ± 0.00d	−1.00 ± 0.00d	−1.00 ± 0.00d	−1.00 ± 0.00c	−1.00 ± 0.00e	−1.00 ± 0.00d	−1.00 ± 0.00c
Ethyl	0.125	−0.14 ± 0.07a	−0.15 ± 0.04a	−0.04 ± 0.05a	−0.10 ± 0.09a	−0.14 ± 0.03a	−0.03 ± 0.05a	−0.15 ± 0.04a	−0.51 ± 0.03a
linolenate	0.25	−0.24 ± 0.03a	−0.16 ± 0.02a	−0.16 ± 0.01ab	−0.29 ± 0.11a	−0.34 ± 0.12ab	−0.16 ± 0.06a	−0.12 ± 0.16a	−0.61 ± 0.03a
	0.50	−0.31 ± 0.09a	−0.14 ± 0.03a	−0.09 ± 0.02bc	−0.33 ± 0.03a	−0.31 ± 0.07ab	−0.15 ± 0.06a	−0.28 ± 0.06a	−0.53 ± 0.07a
	1.00	−0.24 ± 0.03ab	−0.18 ± 0.03a	−0.24 ± 0.00c	−0.39 ± 0.09a	−0.45 ± 0.03b	−0.52 ± 0.01b	−0.73 ± 0.01b	−0.61 ± 0.07a
	2.00	−0.59 ± 0.16b	−0.65 ± 0.02b	−0.59 ± 0.06d	−0.73 ± 0.12b	−0.52 ± 0.03b	−0.69 ± 0.02c	−0.75 ± 0.02b	−0.90 ± 0.03b
Ethyl linoleate	0.125	−0.34 ± 0.09a	−0.14 ± 0.10a	−0.10 ± 0.03a	−0.34 ± 0.00a	−0.24 ± 0.03ab	−0.16 ± 0.05a	−0.20 ± 0.08a	−0.50 ± 0.06a
	0.25	−0.38 ± 0.06a	−0.23 ± 0.06a	−0.18 ± 0.04a	−0.35 ± 0.02a	−0.17 ± 0.06a	−0.21 ± 0.04a	−0.25 ± 0.02a	−0.58 ± 0.08ab
	0.50	−0.38 ± 0.06a	−0.33 ± 0.09ab	−0.13 ± 0.01a	−0.43 ± 0.03a	−0.24 ± 0.09ab	−0.31 ± 0.07a	−0.52 ± 0.04b	−0.58 ± 0.05ab
	1.00	−0.41 ± 0.12a	−0.49 ± 0.08bc	−0.36 ± 0.07b	−0.45 ± 0.08a	−0.38 ± 0.00b	−0.32 ± 0.08a	−0.59 ± 0.03b	−0.70 ± 0.06bc
	2.00	−0.59 ± 0.06a	−0.64 ± 0.05c	−0.64 ± 0.05c	−0.78 ± 0.06a	−0.62 ± 0.03c	−0.59 ± 0.04b	−0.77 ± 0.02c	−0.84 ± 0.02c

*Data are expressed as mean ± standard deviation. Different letters within the same column under same compound signify significant differences at P < 0.05.*

### Physiological Effects of Three Components on *Lolium multiflorum*

**FIGURE 3:**
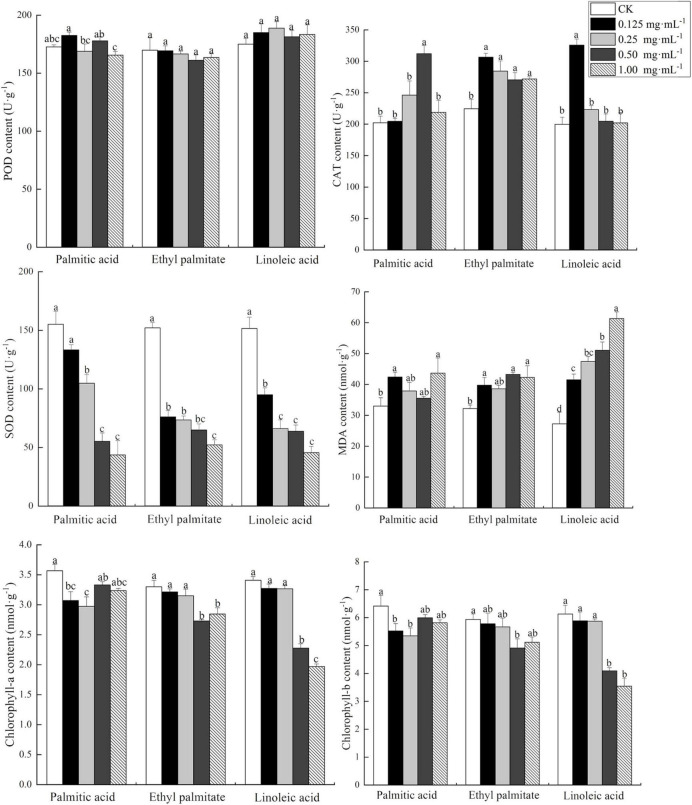
Antioxidant enzyme and chlorophyll properties of fresh *Lolium multiflorum* seedlings affected by three allelopathic substances. SOD, superoxide dismutase; CAT, catalase; POD, peroxidase; MDA, malondialdehyde. Different letters signify significant differences among concentrations of a particular compound at *P* < 0.05.

The enzyme and chlorophyll characteristics of *L. multiflorum* seedlings varied significantly (*P* < 0.05) among concentrations of either PA, EP, or LA (Figure 3). CAT activity in *L. multiflorum* seedlings was higher than CK activity and initially increased and then decreased with increasing concentration of three components. The POD activity of *L. multiflorum* seedlings did not show a clear trend with varying concentrations of the three components. The SOD activity of *L. multiflorum* seedlings significantly declined with increasing concentration of the three components of the sweet potato extract, with inhibition rates of 71.9, 70.0, and 65.7% at a concentration of 1.0 mg mL^–1^ of PA, LA, and EP, respectively (Figure 3). The MDA content of *L. multiflorum* seedlings increased significantly with increasing concentration of the three extract components. Chlorophyll-a and chlorophyll-b contents of *L. multiflorum* seedlings markedly decreased with increasing concentration of all three components (Figure 3).

## Discussion

Sweet potato is a nutritious and medicinal crop that contains abundant polyphenols, vitamin B, calcium, iron, zinc, and proteins (Odake et al., 1994). This plant has demonstrated significant suppression of various weeds through its competitive ability and allelopathic activity (Shen et al., 2014, 2017b,^2018^, ^2019^). Among twenty-one chemical compounds identified in the petroleum ether extract, PA, EL, LAC, EP, and ELL were the most prominent. Harrison et al. (2003) and Chon and Boo (2005) reported that the phenolic compounds such as coumarin, *trans-*cinnamic acid, caffeic acid, and chlorogenic acid were possible allelochemicals in sweet potato. Other phytochemicals such as fatty acids, triterpenes, steroids, alkaloids, quinones, phenols, tannins, flavonoids, saponins, ethyl ester linoleic acid, ethyl ester palmitic acid, *trans-*p-coumaric, and PA with allelochemical potential have been detected in sweet potato (Hernández et al., 2015; Xuan et al., 2016). PA was reported by Xuan et al. (2016), but many compounds in the current study were not previously reported in sweet potato. The variability in chemical composition could be related to the effects of solvents, extracted methods, plant growth stage, and plant species. Moreover, few studies have examined the allelopathic effects of these compounds on other plants, especially invasive alien weeds. PA is also widely found in other crops such as beans, cotton, sunflowers, wheat, rape, and pepper (Lukonge et al., 2007; Zhao et al., 2019). This compound can inhibit plant growth and change SOD, POD activity and proline content of some plants (Zou et al., 2014; Wang et al., 2021), and change the soil nutrient and the rhizosphere microbial composition (Ma et al., 2021).

Allelochemicals are classified as alkaloids, flavonoids, phenols, tannins, cyanohydrins, amino acids, cinnamic acids, peptides, terpenes, ketones, benzoic acid, water-soluble organic acids, fatty acids, unsaturated lactones, quinones, polyacetylenes, coumarin, steroids, or benzoquinones (Macías et al., 2019). Allelopathic intensity depends on the concentration of the extracts, compound types and target species (Xuan et al., 2016; Shen et al., 2018). High extract concentrations usually inhibit seed germination or seedling growth of the target species, but low concentration extracts may actually promote seed germination and seedling growth, showing that stimulatory or inhibitory effect is a function of concentration (Macías et al., 2019). Our results showed that five chemical compounds significantly inhibited germination, plant height and biomass of four invasive weed species, and the effects increased with increasing concentration. Allelopathic effects of the five compounds from the sweet potato extracts were ranked in order with LA exhibiting the greatest inhibition, PA and EP moderate, and EL and ELL having the least inhibitory effects. Biomass and root length were the most inhibited, followed by shoot length, with germination rate showing the least inhibition. Thus, the combined impact of all five compounds could be quite effective in suppressing the invasive weeds of concern, although LA, PA, and EP seem to have much greater potential than the other two allelochemicals. Other research has shown that sweet potato also has allelopathic effects on other plants and crops (Harrison and Peterson, 1986; Walker et al., 1989; Chon and Boo, 2005; Shen et al., 2017b,^2018^; Deng et al., 2020), and different cultivars have various inhibition (Shen et al., 2018). However, these allelopathic effects were mostly determined by different solvent extracts, rather than by pure allelopathic substances.

Allelochemicals have a variety of detrimental effects on the physiology of plant growth and seed germination. Antioxidant enzymes are one of the important reactive oxygen detoxifier systems in plant cells, therefore, an increase in antioxidant enzyme activity can be considered an important defense strategy against oxidative stress (Shi et al., 2006). Oxidative stress can lead to inhibition of the photosynthesis and respiration processes, and thus, plant growth. Lei et al. (2021) found that the mixture of the alkaloids from the desert plant *Sophora alopecuroides* significantly increased the MDA content and POD activity of *Medicago sativa* seedlings, whereas SOD and CAT activity of *M. sativa* seedlings decreased markedly. Ma et al. (2020) reported that two allelochemicals benzoic acid and cinnamic acid from invasive plants reduced growth and chlorophyll content in *Chrysanthemum coronarium*. The effects on *C. coronarium* growth were shown to be influenced by increased antioxidant activity by SOD and CAT, and modified MDA content, with POD activity declining indicating extreme sensitivity of POD to the allelochemicals. Despite the increased antioxidant activity by SOD and CAT, once too much reactive oxygen had accumulated, plant tissue damage resulted (Ma et al., 2020). Our study found that CAT, MDA, and POD contents of *L. multiflorum* were increased by three allelochemicals LA, PA, and EP, whereas SOD content decreased. Higher rates of photosynthesis connected to higher chlorophyll content can lead to increased growth rates, biomass accumulation and overall production (Shen et al., 2019). The chlorophyll-a, chlorophyll-b and carotenoid content of *Macrotyloma uniflorum* showed decreasing effects upon exposure to *Mikania micrantha* extracts (Jali et al., 2021). In the present study, we found that both the chlorophyll-a and chlorophyll-b content of *L. multiflorum* were greatly reduced with increasing concentration of PA, LA, and EP. Thus, it is clear that these three compounds change the antioxidant enzymes SOD, POD, CAT, and MDA reducing chlorophyll content in *L. multiflorum*, thus affecting the germination rate and seedling growth of *L. multiflorum*.

Sweet potato has been recognized as a very competitive crop against certain weeds because of its rapid growth, rapid canopy formation, and its ability to reproduce asexually (Shen et al., 2014, 2015, 2019). The crop has been shown to significantly reduce the density, frequency, cover, and importance value of *B. pilosa* and *G. parviflora* in sweet potato fields, through both competitive ability and allelopathy (Shen et al., 2014, 2017b). The current study demonstrated that sweet potato possesses specific allelochemicals that suppress the seed germination and seedling growth of the four invasive weed species. Therefore, sweet potato has considerable potential to provide an effective ecological management tool for weed management in the field.

## Conclusion

Palmitic acid, EL, LA, EP, and ELL were identified as five major compounds with allelochemical potential in a petroleum ether extract of sweet potato. These compounds exhibited strong inhibitory activity against four invasive alien weeds *B. pilosa*, *G. parviflora*, *L. multiflorum*, and *P. minor*. The allelopathic effects were ranked in order from LA (greatest), PA and EP (moderate), and EL and ELL (least). Thus, LA, PA, and EP represent the most promising allelochemicals for suppression of invasive weeds. When the impacts of these three strongest allelochemicals were tested on the antioxidant activity in *L. multiflorum*, altered levels of CAT, MDA, POD, and SOD reduced the germination and seedling growth of *L. multiflorum*, further confirming the multitarget impacts of these particular allelochemicals. More studies of the biosynthetic pathways of allelopathic compounds, potential for particularly allelopathic genotypes and the regulatory mechanisms of sweet potato germplasm resources are needed to better inform the use of sweet potato as an allelopathic crop.

## Data Availability Statement

The original contributions presented in the study are included in the article/supplementary material, further inquiries can be directed to the corresponding author.

## Author Contributions

SS conceived and designed the experiments, performed the experiments, analyzed the data, and wrote the draft of the manuscript. GM performed the experiments and analyzed the data. GX, DL, GJ, SY, AC, and LW performed the experiments. DC analyzed the data and wrote the draft of the manuscript. FZ conceived and designed the experiments and performed the experiments. MY reviewed the draft. All authors read and approved the final manuscript.

## Conflict of Interest

The authors declare that the research was conducted in the absence of any commercial or financial relationships that could be construed as a potential conflict of interest.

## Publisher’s Note

All claims expressed in this article are solely those of the authors and do not necessarily represent those of their affiliated organizations, or those of the publisher, the editors and the reviewers. Any product that may be evaluated in this article, or claim that may be made by its manufacturer, is not guaranteed or endorsed by the publisher.
